# Plasticity mechanisms in HfN at elevated and room temperature

**DOI:** 10.1038/srep34571

**Published:** 2016-10-06

**Authors:** Katherine Vinson, Xiao-Xiang Yu, Nicholas De Leon, Christopher R. Weinberger, Gregory B. Thompson

**Affiliations:** 1 The University of Alabama, Department of Metallurgical & Materials Engineering, 245 7th Avenue 360 HM Comer Hall, Tuscaloosa, AL 35487, USA; 2 Drexel University, Department of Mechanical Engineering & Mechanics, 3141 Chestnut Street, 115 Randell Hall, Philadelphia, PA 19104, USA

## Abstract

HfN specimens deformed via four-point bend tests at room temperature and at 2300 °C (~0.7 T_m_) showed increased plasticity response with temperature. Dynamic diffraction via transmission electron microscopy (TEM) revealed ⟨110⟩{111} as the primary slip system in both temperature regimes and ⟨110⟩{110} to be a secondary slip system activated at elevated temperature. Dislocation line lengths changed from a primarily linear to a curved morphology with increasing temperature suggestive of increased dislocation mobility being responsible for the brittle to ductile temperature transition. First principle generalized stacking fault energy calculations revealed an intrinsic stacking fault (ISF) along ⟨112⟩{111}, which is the partial dislocation direction for slip on these close packed planes. Though B1 structures, such as NaCl and HfC predominately slip on ⟨110⟩{110}, the ISF here is believed to facilitate slip on the {111} planes for this B1 HfN phase.

Ultrahigh Temperature Ceramics (UHTC’s) are materials known for their high melting temperatures (T_m_), hardness, moderate oxidation resistance, and having a range of thermomechanical behaviors^1^,^2^,^3^,^4^,^5^. Their exceptional properties have been attributed to strong covalent bonding between a refractory metal atom and a nonmetal atom that compose their unit cells^6^,^7^. For these reasons UHTC’s have been an area of active research for wear resistant cutting tools^8^, thermal protection systems in atmospheric reentry vehicles, and sharp leading edges or nose tips in hypersonic vehicles^9^. To fully develop the strength of these materials for load bearing applications, one must understand the intrinsic mechanisms of deformation^10^.

Many UHTC compounds exhibit a B1, or rocksalt, structure. This crystal arrangement can be described as two interpenetrating face centered cubic (FCC) sublattices. Being an FCC-based structure, one could assume that 1/2〈110〉{111} would be the dominate slip mechanism based on simple geometric arguments. The closed packed directions, 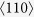, along with the close packed {111} planes provide the largest inter-planar spacing and therefore yield the shortest Burgers vector which minimizes strain energy on those planes for slip^11^. However, ionic B1 structures, like NaCl, often slip, not on {111} planes, but on {110} planes, which is attributed to the dominance of ionic bonding in these materials. This simple example demonstrates that the nature of bonding can have a dramatic impact on the operating slip systems. This becomes even more complicated in the mixed covalent-metallic-ionic bonding present in B1 structured transition metal carbides and nitrides in which slip has been reported to occur on both {110} and {111} B1 planes^12^.

The primary slip systems in TiC and HfC (group IVB transition metal carbides) have been experimentally identified as the 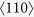
{110} type under microindentation at room temperature^13^,^14^. This slip, however, can change at elevated temperatures. TiC has been shown to activate 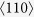
{111} slip above its brittle-to-ductile (BTD) transition temperature, having highly mobile mixed dislocations^13^,^15^. To the authors’ knowledge, no dislocation studies have yet been reported on HfC above its BTD transition temperature. For the group VB transition metal carbides, the TaC slip system has been rigorously iestigated and determined to be 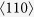
{111} at room and elevated temperatures^14^,^16^,^17^,^18^. The difference in slip planes ({110} vs. {111}) between HfC and TaC offers insights into the detailed role that bonding plays in regulating deformation mechanisms in the B1 structure. Since both carbides have the same crystal symmetry as well as similar melting points near 4000 °C, one could be perplexed about the mechanisms that regulate slip.

To address this issue, De Leon *et al*. performed a series of first principal simulations to investigate the generalized stacking fault energy (GSFE) on these surfaces^18^. For the perfect slip direction in HfC and TaC, it was found that 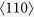
{110} curves were lower in energy than 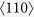
{111}. However, in FCC-based structures, slip on the {111} surfaces usually involves the dissociation of the perfect dislocation into two partial dislocations of the type 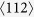
{111}, commonly referred to as Shockley partial dislocations. By considering this dissociation reaction and the full GSFE surface, it was found that TaC has an intrinsic stacking fault (ISF) on the {111} surface where HfC does not. This ISF provides a local energy minimum and allows TaC to prefer slip on the {111} rather than {110} planes. The presence of the ISF is also believed to affect phase stability by promoting stabilization of stacking fault phases that readily form in transition metal group VB carbides but are absent in the transition metal group IVB carbides^19^. De Leon *et al*. attributed the stabilization of the ISF to TaC having a more metallic character than HfC which is related to the available number of valence electrons of the transition metal in the B1 bonding environment. Sangiovanni *et al*. even suggested that alloying group IVB carbides with 50% group VB or VIB transition metals would make these carbides “supertough” because of an increased concentration of valence electrons^20^. Though they did not expound on the mechanism, we believe that to be correct as the more metallic the bond becomes, the more likely the ISF can exist in the B1 structure based on the findings of De Leon *et al*.^18^.

Similar slip concepts have been proposed in the B1 structures TiC and TiN, where TiC will behave similar to HfC as noted above. However, in the nitride, the nonmetal species now has a higher density of valence electrons, creating a similar bonding environment as the group VB transitional metal carbides^21^. In the computational work of Zhang *et al*., they noted that the bonding in TiN was predicted to be less directional than that of TiC and an ISF is stabilized in TiN while there is none in TiC. To date, there has been no experimental work done to validate this model^21^. Additionally, Li and Howe reported that the slip of ZrN (a group IVB transition metal nitride) was dominant on the 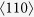
{111}, with slip being predicted to become more favorable on the {111} surfaces at substoichiometric compositions^22^.

To date there have not been any rigorous, self-contained studies to determine, both experimentally and computationally, the energy hierarchy for deformation in the group IVB transition metal nitrides. Such a study would provide insights into the global behavior that governs slip between B1 ceramic materials. As suggested by the more recent findings of De Leon *et al*.^18^, it would seem that in systems with a {111} ISF, slip would be preferred on those planes. If such an ISF does not exist, then slip would be favored on {110} planes. Moreover, simple comparisons of valence shell states could provide the first approximation of ISF stability, although more rigorous models are desired. To test the hypothesis that the presence of an ISF on {111} planes enables these planes to dominate slip in B1 compounds, we have performed an experimental and computational investigation in the HfN system.

## Results

### Computational comparison of slip systems

**Figure 1:**
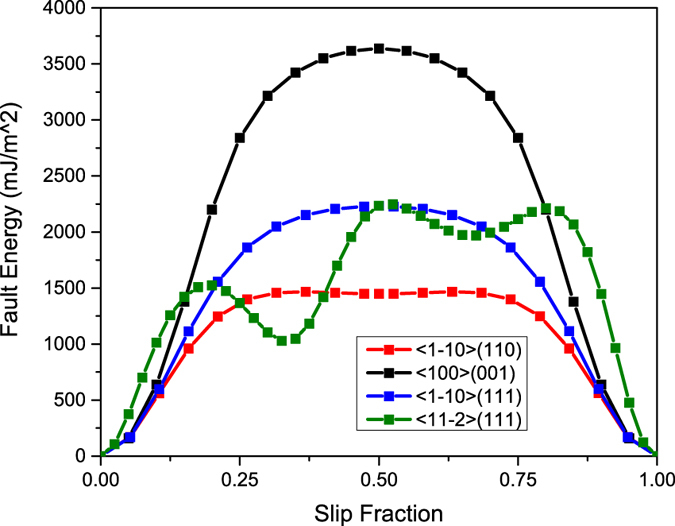
GSF energies in each HfN slip system.

Considering the families (groups) to which Hf and N belong, we suspect that an ISF would be present based on the total number of valence electrons per formula unit, which is equal to 9. The GSFE curves are plotted in in Fig. 1. The first feature that can be gleaned from these curves is that indeed, a stable ISF exists in HfN as suggested from its valence configuration. This is seen as the local minimum near 0.33 fraction shift for the 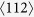
{111}, and is the lowest energy of all the slip systems simulated. The ISF would allow the perfect dislocation to split into partials, increasing the width of the dislocation and hence reducing the barrier for motion. This makes the {111} a more favorable plane for slip. Interestingly, near 0.65 fractional shift, another minor minimum is noted on the same curve, implying some bonding is created as the hafnium and nitrogen atoms sit atop one another, a feature not usually observed in GSFE surfaces. These fault energies and their hierarchy are comparable to prior tantalum carbide work^18^, implying dislocation densities, shapes, etc. will be consistent between these two classes of UHTCs.

### Slip Behavior

**Figure 2:**
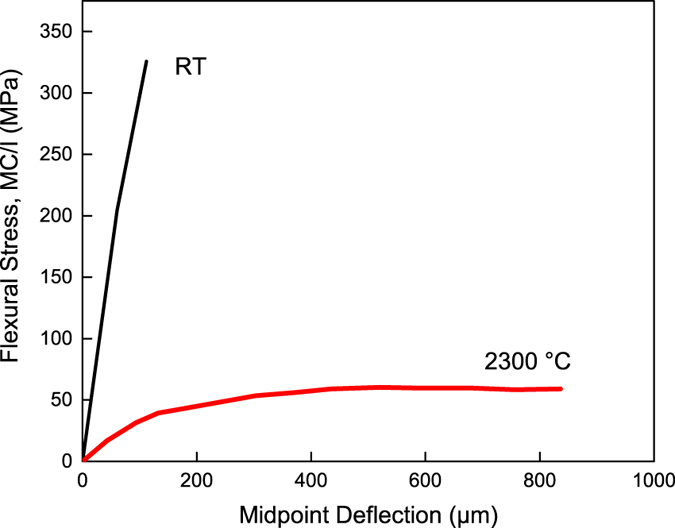
Mid-point deflection in four-point bend test of HfN at room temperature and 2300 °C.

**Figure 3:**
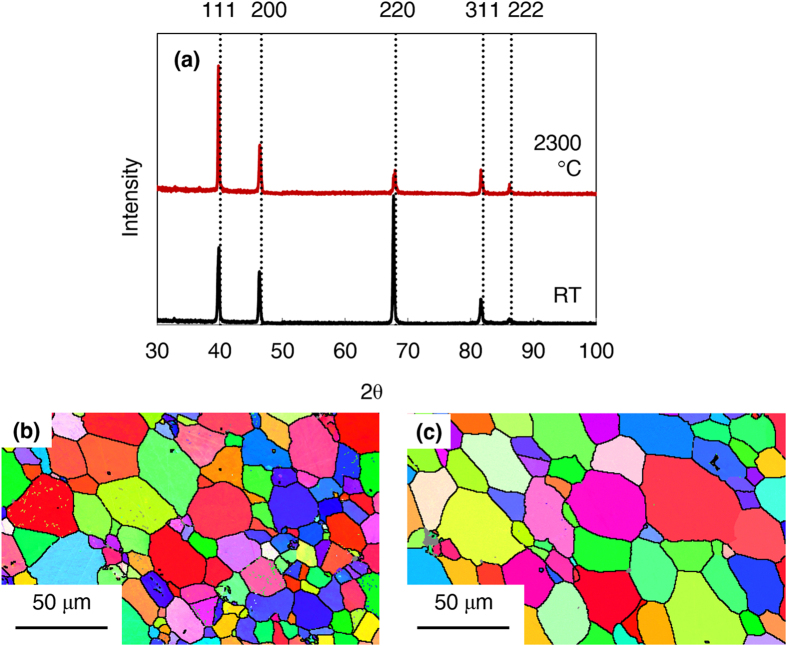
Phase characterization of post-tested bars (**a**) Normalized peaks collected by X-ray diffraction of post-tested bars at room temperature and 2300 °C reveal bars are polycrystalline single phase HfN. SEM-EBSD showing grain size and shape of post tested HfN bars at (**b**) room temperature and (**c**) high temperature.

Figure 2 is the load verses deflection curve of HfN at room temperature and 2300 °C. Both bars showed some plasticity, with the elevated temperature being more plastic. Because mechanical response can be a function of grain size, texture, phase, and composition, XRD, EBSD, and LECO analysis was carried out to determine if one or more of these factors contributed to the change in flexural strength. An XRD scan, Fig. 3(a), detected diffraction from only the B1 HfN phase in both the room and elevated temperature samples. Note that no oxide peaks were present, providing evidence that the Ar cover gas used during the elevated temperature test was sufficient to prevent significant oxidation. The EBSD scans of each testing condition, given in Fig. 3(b,c), established that both samples have a polycrystalline grain structure with no obvious texture. The grain size was found to be 15.2 ± 1.39 μm at room temperature and 19.7 ± 8.58 μm at 2300 °C. The average grain size did coarsen by approximately 30% between the two experiments, but the difference was minimal, evident by the relative error values between the two runs. LECO analysis revealed the bar tested at room temperature had 50.4 ± 0.10 at% N and 1.94 ± 0.014 at% O while the bar tested at 2300 °C had 50.4 ± 0.066 at% N and 2.07 ± 0.052 at % O, confirming that the bars were stoichiometric and there was no loss of nitrogen during testing. Collectively, the near equal grain size, grain shape, polycrystalline texture, equivalent phase, and consistent chemical composition indicate that these microstructure factors could not account for the flexural strength differences. This raises the important question of how plasticity contributed to the differences in the deformation.

**Figure 4:**
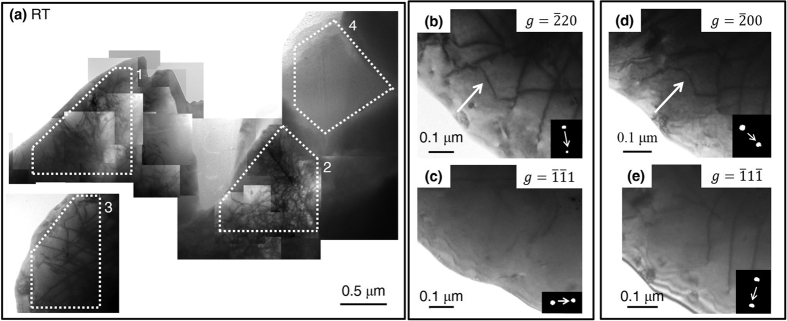
TEM of HfN deformed at room temperature (**a**) Compiled TEM images of dislocations in HfN under various two-beam conditions. The white arrows indicate a dislocation whose Burger’s vector, *b* = *a*/2 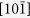, lies on the (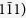 slip plane (**b**) imaged under a visible two-beam condition *g* = 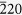, (**c**) the corresponding invisible condition has diffracted beam *g* = 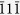. (**d**) The same dislocation is imaged under the visible condition *g* = 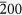, (**e**) and its corresponding invisible condition is *g* = 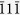.

HfN deformed at room temperature was imaged under various two-beam conditions and a collection of those images is given in Fig. 4(a). In general, there are regions with entangled, interacting dislocations (areas 1 and 2), regions with linear, well-spaced dislocations forming an ordered network (area 3), and regions with no dislocation activity at all (area 4). This structure difference is due to the complex loading conditions involved in four-point bending where parts of the bar are in compression and parts in tension as well as a change in grain orientation with respect to the loading direction causing each grain to respond differently to the load. The dislocation density averaged from the two regions with entangled networks (areas 1 and 2) is approximately 6 × 10^14^ m^−2^ and the dislocation density in the region with a linear arrangement (area 3) is approximately 2 × 10^14^ m^−2^. The average dislocation density over the entire imaged area show in Fig. 4(a) is 1.6 × 10^14^ m^−2^.

Dynamical diffraction conditions for visible and invisible imaging of similar dislocations in the room temperature specimen are provided in Fig. 2(b–e). The arrows in these micrographs denote the dislocation of interest. For the sake of brevity, only one identified dislocation is reported here but other dislocations within this foil showed the same character, type, and equivalent slip plane family. The Burgers vector was determined to *b* = *a/2* 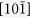 lying on the (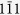) plane.

**Figure 5:**
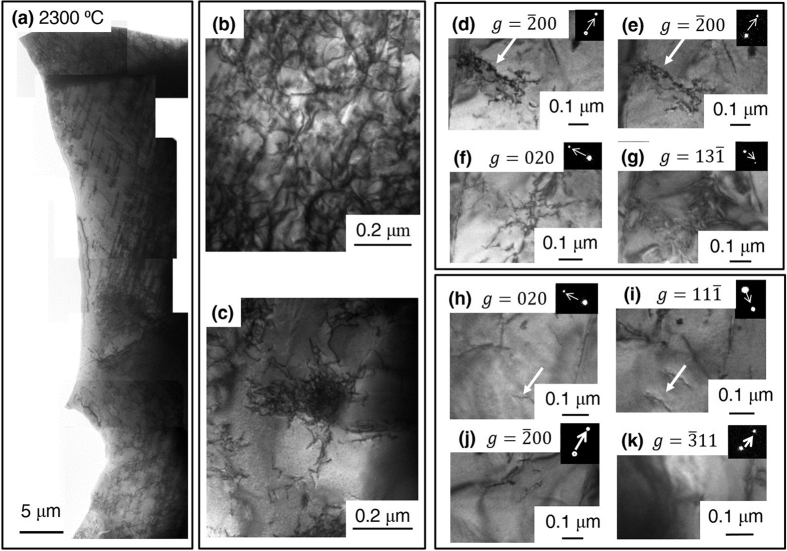
TEM images of HfN deformed at 2300 °C (**a**) Compiled TEM images of dislocations under various two-beam conditions. (**b,c**) Two-beam images of networking dislocations (**d,e**) Two-beam visible conditions, where the dislocations of interest are indicated by white arrows of dislocations, with b = *a*/2 [101] on the 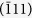 slip plane piling up at a grain boundary under the two-beam condition *g* = 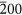. The corresponding invisible two-beam conditions, (**f**) *g* = 020 (**g**) *g = *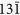. A second set of HT dislocation with b = *a*/2 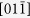 on the (011) slip plane with corresponding two-beam conditions in the inset. The dislocation of interest is indicated again with white arrows in the two visible conditions (**h**) with *g* = 020 and (**i**) *g* = 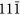 and the corresponding invisible conditions (**j**) *g* = 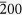 and (**k**) *g* = 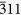.

TEM micrographs for the 2300 °C specimen are reported in Fig. 5. Here, the dislocations form networks; and the dislocation density averaged over areas shown in Fig. 5(a,b) is ~6.4 × 10^13^ m^−2^. Similar to room temperature deformation, in the bar deformed at elevated temperature, there are distinctly different dislocation microstructures. There are regions where dislocations are linear and ordered with a dislocation density of ~2.3 × 10^13^ m^−2^ (Fig. 5a), regions with dense forests ~5.1 × 10^14^ m^−2^ (Fig. 5b), and regions where the dislocations are so dense that their densities cannot be counted because of their overlapping contrast (Fig. 5c). Unlike the room temperature specimen, at elevated temperature, there were no observed areas without some dislocation activity. The feature shapes of these dislocations reveal highly mobile behavior which would be indicative of the increased plasticity noted in the deflection curve of Fig. 1. Figure 5(d–g) reveal the visible and invisible conditions for one particular dislocation, indicated with an arrow. This was identified to have a character of *b* = *a/*2 [101] gliding on the (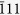); which is consistent with room temperature slip. Further investigations of another dislocation, Fig. 5(h–k) revealed the dislocation of interest had a Burger’s vector, *b* = *a/*2 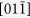, gliding on the (011) slip. Hence, at elevated temperature, multiple slip systems have been confirmed to be active.

## Discussion

First principal calculations of GSFE curves confirmed that, like TaC, HfN has a stabilized ISF (Fig. 1). This ISF in HfN, where there is not one in HfC, is attributed to HfN having one more valence electron than HfC (8 in HfC vs. 9 in HfN). The primary slip system was experimentally determined to be the 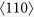
{111} system (Figs 4 and 5). The 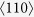
{110} slip system has a maximum GSFE of 1,468 mJ/m^2^, while the 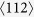
{112} slip system has a local energy well at 1,028 mJ/m^2^ where the Shockley partial is stable. Both these energies are lower than the slip energy of the 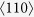
{111} system experimentally determined, 2,227 mJ/m^2^.

To rationalize why these partial dislocation types having the lowest GSFE were not observed, one must consider the splitting width between each partial. It is known that B1 carbides and nitrides generally have a larger GSFE than other fcc materials whose partial dislocations are widely spaced. For example, the calculated GSFE on the {111} plane has been reported to be less than 200 mJ/m^2^ along the 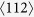 and less than 700 mJ/m^2^ along the 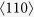 plane in gold, silver, copper, and aluminum^23^. Additionally, in a study on austenitic stainless steels, Lu *et al*. determined experimentally and computationally that the stacking fault energy (SFE) is less than 30 mJ/m^2^ 24. In comparison, the GSFE in carbides and nitrides has been calculated to be between 1000 and 4000 mJ/m^2^ 21,25,26. The GSFE in the carbides and nitrides, which can be one or two orders of magnitude larger than that of fcc metals, will keep the partial dislocations too close together to resolve by conventional bright field TEM imaging methods. Because of the large SFE and stable ISF, it is suspected that the dislocations characterized as *a/*2 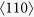
{111} are *a/*6 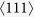
{111} with the Shockley partials too close together to resolve. Attempts using weak-beam dynamical diffraction did not provide sufficient image resolution to see this splitting. Regardless, this experimental finding supports our hypothesis that the ISF stabilizes {111} slip and that the number of valence electrons is responsible for regulating slip.

The difference in flexural deflection with increasing temperature in HfN is evidenced by the change in slope of the deflection curve between the bar deformed at room temperature and the one deformed at 2300 °C. The bar tested at room temperature has a deformation curve with a steep slope, indicating elastic deformation and does not appear to enter a plastic regime prior to failure, which is the expected mechanism of failure in brittle materials. Contrastingly, the bar deformed at 2300 °C has a flat curve indicating it experienced extensive plasticity and the existence of a BTD transition observed in many other UHTC’s. There was no change in phase or chemistry at elevated temperature, confirmed by XRD (Fig. 2a) and LECO chemical analysis. There was also minimal, self-consistent grain growth (Fig. 2b,c). Since the 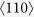
{111} slip systems identified in this study has the five independent slip systems necessary to accommodate deformation in polycrystalline materials according on the von Mises criterion^27^, the BTD transition is not controlled by the activation of new slip systems. Rather, it must be governed by either the mobility or nucleation of dislocations. Our room temperature bending experiments demonstrate that a sufficient number of dislocations already existed to support bulk plastic deformation, i.e. dislocation densities at higher temperatures are not markedly different. This suggests that the BDT is controlled by dislocation mobility. Dislocations in the specimens deformed at room temperature frequently exhibited long straight dislocations, suggesting high lattice friction, which supports the notion that the lattice resistance is large in these materials and regulates deformation. In contrast, the elevated temperature dislocations appeared more curved and tended to form dislocation substructures, similar to deformation in metals. Kim *et al*. reported similar dislocation behavior in tantalum carbides^16^, which have the same slip planes and stabilized ISFs, suggesting that dislocation mobility regulates slip in B1 transition metal carbides and nitrides within the B1 structure.

In contrast, the B1 transition metal carbides and nitrides which do not exhibit ISF’s, deform via slip on {110} planes and are more brittle^14^,^28^. At elevated temperatures, i.e. in TiC, the slip system changes from 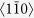
{110} to 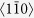
{111} above 800 °C, suggesting thermal energy is necessary to active this additional, higher energy slip system and to accommodate plastic deformation^15^,^28^,^29^. This may indicate that the BTD transition in these materials may be controlled by the number of available slip systems, although further work in this area is warranted. The presence of the 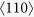
{110} slip, observed at elevated temperature for HfN, similarly suggests that sufficient thermal energy is needed to activate the next higher energy slip system.

In addition to a secondary slip system being activated, at elevated temperatures, thermal energy would cause dislocations to cross-slip and experience diffusional climb, making them more mobile. While the dislocation density in the room temperature specimen is slightly higher than that of the elevated temperature one (1.6 × 10^14^ m^−2^ vs. 6.4 × 10^13^ m^−2^), at room temperature, there were regions with no dislocation activity. Conversely, the elevated temperature specimen had relatively consistent dislocation density throughout. Though, in some specific regions, larger densities were noted as evident by dislocation entanglement, Fig. 5(c), where the dislocation density could not be calculated because of overlapping contrast from the individual dislocations. Such dense areas, if counted, would likely cause the density in the elevated temperature specimen to be even larger than that of the room temperature.

## Conclusion

Two HIP’d HfN bars were tested in four-point bending, one at room temperature and one at 2300 °C. The bar deformed at room temperature exhibited limited plasticity while the one deformed at elevated temperature showed significant plasticity. XRD, SEM-EBSD, and LECO analysis revealed that the phase composition, grain shape, size, and stoichiometry remained constant between the two tests, eliminating these issues as sources for enhanced plasticity. Dislocations in each post-tested bar were characterized with TEM and the dynamical ***g ***∙ ***b ***×** *****u*** imaging method. At room temperature, the active slip system was indexed as the 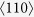
{111} system. At elevated temperature, two active slip systems were identified, 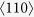
{111} and 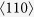
{110}. The increased temperature is believed to provide sufficient thermal energy to activate a secondary slip system. The microstructure of dislocations in the bars both showed some level of low-energy network formation. At room temperature, the dislocations were either long and straight, or tangled; both configurations are indicative of lower mobility. At elevated temperature, the dislocations were curved, intertwined into thick bunches, or beginning to form subgrain networks.

The dislocation content and behavior observed in the micrographs suggest that the BTD transition is controlled by dislocation mobility. This is because the number of dislocations present at low temperatures is sufficient to carry bulk plastic deformation and the active slip systems, 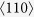
{111}, are sufficient to support the von Mises criteria for general plastic deformation. The presence of 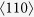
{110} slip is attributed to the higher thermal activation of a secondary slip system, similar to the activation of 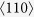
{001} slip in aluminum at higher temperatures^27^.

Using DFT, the slip system energy hierarchy was revealed. HfN had an ISF on the {111} plane which enabled slip on the close packed planes. This result, similar to recent report explaining slip differences between TaC and HfC^18^, suggests materials with this ISF are able to circumvent 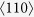
{111} slip commonly seen in B1 ceramic materials and instead propagate slip on the {111} planes. Predictions in other systems with similar characteristics could be made by identifying the transition metal family’s available valence electrons as the main indicator for forming an intrinsic fault for materials that share the B1 structure.

## Methods

### Computational Methods

To determine the GSFE curves in HfN, first principal density functional theory (DFT) calculations were carried out with the plane-wave based Vienna ab initio simulation package (VASP)^30^,^31^ using the projector augmented wave method^32^,^33^ and the generalized gradient approximation in the parametrization by Perdew *et al*.^34^. Plane waves were included up to a cutoff energy of 600 eV, which yielded convergent results. The convergence accuracy of the total energy was chosen as 10^−5^ eV in the relaxation of electronic degrees of freedom. The structures of the models were relaxed until the maximum force was less than 0.02 eV/Å. The Monkhorst–Pack scheme was used for k-points sampling^35^. To eliminate the interactions of parallel faults, a vacuum region of 15 Å was introduced to separate the parallel faults. The supercells contain 20 layers along the [001] direction, 32 layers along the [110] direction, and 30 layers along the 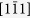 direction. The top and bottom two layers were fixed during relaxation to avoid surface reconfiguration.

To calculate the planar fault energies, displacement in the supercells was introduced on the (001) plane along the [010] direction, on the 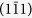 plane along 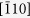 and 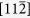 directions and on the (110) plane along 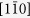 direction. The atoms were allowed to relax normal to the slip plane, while the position of atoms in the plane was fixed to simulate the rigid slip.

### Fabrication and flexural testing

The operative slip systems were experimentally determined by a series of room and elevated temperature flexural tests of HfN. The HfN flexural bars were prepared from stoichiometric HfN powders fabricated at Exothermics, Inc. The powders were cold compressed into a Ta canister, evacuated, sealed and then hot isostatically pressed (HIP’d) at 1800 °C/200 MPa/4 hours. Post-HIP, the Ta canister was mechanically cut from the HfN billet and the billet was machined into 3 mm × 4 mm × 45 mm test bars. These bars were then mechanically loaded in four-point flexural bending apparatus under the ASTM standard C 1211–02^36^. The bars were tested at room temperature (23 °C) and near 2300 °C (~0.7 T_m_^37^). For the elevated temperature, the flexural unit was encased in a graphite element furnace with Ar flowing over the bar to avoid oxidation during the test.

### Phase and Composition

A small section from the end of each post-tested bar, far from the maximum midpoint deflection, was cut with a diamond wafering blade and crushed into a fine powder for X-ray Diffraction (XRD) phase analysis. A Bruker Discovery D8 General Area Diffraction Detector System (GADDS) with a Co Kα1 radiation source, λ = 1.78896 Å, operated at 45 keV and 40 mA was employed to collect these XRD patterns to confirm the crystal structure. In addition, one half of each post-tested bar was reserved for thermo-combustion (LECO) chemical testing to determine the stoichiometry of the bar.

Another small section of the bar, far from the maximum midpoint deflection, was mounted in PolyFast resin, a thermosetting conductive epoxy with conductive carbon filler, for Scanning Electron Microscopy (SEM) imaging. SiC grinding paper was used to polish the mounted samples to 1200 grit and then they were further polished using 9 μm and 3 μm diamond suspension. Finally, the samples were polished with a 0.5 μm silica suspension using a Vibromet^TM^ polisher for approximately 12 hours. Grain size determination was performed by Electron Backscattered Diffraction (EBSD) using a JEOL 7000F field emission microscope operated at 20 keV and 80 μA. The EBSD detector used 4 × 4 binning, low gain, and a step size of 0.44 μm and 0.19 μm for the room temperature and elevated temperature samples respectively.

### Transmission Electron Microscopy (TEM)

To make thin Transmission Electron Microscopy (TEM) foils from the region of maximum deflection, a cross-section from the midpoint deflection was sectioned with a diamond blade. The sections were then ground to 300 μm thickness with 1000 grit SiC grinding paper where upon a Fischione Instruments Model 170 ultrasonic drill with SiC abrasive slurry was used to cut a 3 mm disc. The disc was then further ground to approximately 100 μm with 1200 grit SiC grinding paper. The disc was then dimpled to 7 μm with a Fischione Instruments 200 dimpling grinder using a 6 μm diamond paste polish under a rotating 15 mm steel wheel. Finally, a perforated hole, where electron transparency regions resided near the edges of the hole, was argon ion milled using a Gatan Precision Ion Polishing System (PIPS^®^) in three steps. The PIPS^®^ milled the constantly rotating disc at 4.5 keV with guns at 7° on the top and bottom to achieve a rupture in the center of the disc. Once there was a visible hole, the disc was further milled at 2.5 keV with the guns aimed at 4° top, 2° bottom for one hour and then ion cleaned at 0.5 keV with a gun setting of 4° top and 2° bottom for one hour.

A 200 keV FEI F20 Tecnai (S)TEM was used to characterize foils. The dislocation identity was determined using the dynamical ***g *****∙ *****b *****× *****u*** characterization method, where **g** is the diffraction vector, ***b*** is the Burgers vector, and ***u*** is the line sense of the dislocation^38^. Large field of views were also collected under various two-beam conditions and compiled using Gimp GNU Image Manipulation Program. Dislocation densities were calculated by a method presented by R. K. Ham^39^ using (equation 1), where *L* is the total length of a line marked on the image, *N* is the number of intersecting dislocations, and *t* is the thickness of the foil, which was estimated to be 100 nm.


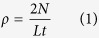


## Additional Information

**How to cite this article**: Vinson, K. *et al*. Plasticity mechanisms in HfN at elevated and room temperature. *Sci. Rep.* **6**, 34571; doi: 10.1038/srep34571 (2016).
